# The Role of Pancreatic Stone Protein (PSP) as a Biomarker of Pregnancy-Related Diseases

**DOI:** 10.3390/jcm12134428

**Published:** 2023-06-30

**Authors:** Romana Brun, Ladina Vonzun, Benjamin Cliffe, Nora Gadient-Limani, Marcel André Schneider, Theresia Reding, Rolf Graf, Perparim Limani, Nicole Ochsenbein-Kölble

**Affiliations:** 1 Department of Obstetrics, University Hospital of Zurich, Frauenklinikstrasse 10, CH-8091 Zurich, Switzerland; 2 Faculty of Medicine, University of Zurich, Rämistrasse 71, CH-8091 Zurich, Switzerlandtheresia.reding.graf@usz.ch (T.R.); perparim.limani@usz.ch (P.L.); 3 Department of Obstetrics and Gynaecology, Cantonal Hospital Baden, 5404 Baden, Switzerland; nora.gadient@hotmail.ch; 4 Swiss Hepatopancreatobiliary Laboratory, Department of Surgery & Transplantation, University Hospital Zurich, Raemistrasse 100, CH-8091 Zurich, Switzerland

**Keywords:** PSP, pancreatic stone protein, preterm premature rupture of membranes, pre-eclampsia, HELLP syndrome, COVID-19, intraamniotic infection

## Abstract

*Background:* Pancreatic stone protein (PSP) is a biochemical serum marker that contains levels that are elevated in various inflammatory and infectious diseases. The role of PSP in the diagnosis of these diseases seems to be more important compared to clinically established biochemical serum markers in discriminating the severity of the same diseases. Standard values for PSP in pregnant women in relation to gestational age have been reported recently. Additionally, increased PSP levels have been observed to be associated with renal dysfunction in pregnant women. The aim of this study is to evaluate the diagnostic role of PSP in pregnancy-related diseases, such as pre-eclampsia (PE), hemolysis-elevated liver enzymes, and low platelet (HELLP) syndrome. In addition, the study aims to assess its diagnostic role in inflammation-triggered diseases as preterm premature rupture of membranes (PPROM) or COVID-19-positive pregnant women. *Materials and Methods:* In this single-centred prospective study performed at a tertiary university hospital between 2013 and 2021, we included 152 pregnant women who were diagnosed with either PE, HELLP syndrome, or PPROM. In December 2020, in the context of the COVID-19 pandemic, the Independent Ethics Committee (IEC) approved an amendment to the study protocol. Depending on the underlying disease, single or serial-serum PSP measurements were assessed. These PSP values were compared to PSP levels of women with normal pregnancies. *Results:* Pregnant women diagnosed with pre-eclampsia or HELLP syndrome had significantly increased PSP values (mean 9.8 ng/mL, SD 2.6) compared to healthy singleton pregnant women (mean 7.9 ng/mL, SD 2.6, *p* ≤ 0.001). There was no difference in serum PSP in pregnant women with PPROM compared to women with uncomplicated singleton pregnancies (mean in PPROM: 7.9 ng/mL; SD 2.9 versus mean in healthy pregnancies: 7.9 ng/mL; SD 2.6, *p* = 0.98). Furthermore, no difference in the PSP values in women with or without intra-amniotic infection was observed (infection: mean 7.9 ng/mL; SD 2.8 versus no infection: mean 7.8 ng/mL; SD 3, *p* = 0.85). The mean value of PSP in COVID-19-infected women during pregnancy (8.5 ng/mL, SD 2.3) was comparable to healthy singleton pregnancies (mean 7.9 ng/mL, SD 2.6), *p* = 0.24. *Conclusions:* The novel serum biomarker PSP is significantly upregulated in pregnant women with pre-eclampsia and HELLP syndrome. Our observations call for the further evaluation of PSP in randomized controlled clinical trials to demonstrate the actual role of PSP in pregnancy-related diseases and whether it may provide new approaches for the management and discrimination of the severity of these gestational conditions.

## 1. Introduction

Pancreatic stone protein (PSP) is a protein physiologically produced mainly in the pancreas and the gastrointestinal tract. Experimental and clinical trials demonstrated that serum PSP levels are elevated in various inflammatory or infectious diseases [1,2,3]. Increased levels of PSP have been detected in polytrauma patients developing sepsis [1,2,4]. Additionally, PSP is supportive in predicting severity of peritonitis and mortality in intensive care unit patients [5]. Moreover, PSP has a higher diagnostic performance for the diagnosis of sepsis compared to other established biochemical biomarkers, such as procalcitonin (PCT) and C-reactive protein (CRP) [6]. In combination with PCT, PSP is a valuable biomarker in early onset sepsis (EOS) [7]. In acute burn care, PSP differentiates between septic and non-septic patients [8]. PSP and Regenerating (REG) family proteins have been revealed as being identical proteins and occur both in the urine and renal calculi of healthy individuals [9].

Furthermore, PSP plays a role in the beta–cell regeneration process of the pancreas [10]. In the same preclinical experiments, the expression of PSP has been shown to be induced by stress conditions without any pancreatic inflammation [11]. Recently, a systematic review with meta-analysis evaluated the accuracy of PSP for the diagnosis of infectious diseases among hospitalized patients [12]. They calculated for the PSP a cut-off value of 44.18 ng/mL (presence of infection versus absence of infection) and confirmed the significant better performance of PSP compared to CRP [12].

So far, there are no studies evaluating the role of PSP as a marker of infectious and inflammatory diseases in pregnant women. It has been reported in 595 pregnant women that high PSP concentrations were closely associated with renal dysfunction in pregnant women [9]. We have recently published reference values of PSP in pregnant women with physiological pregnancies. They could demonstrate that these values were comparable to published standard values in non-pregnant patients [13].

This insight blazes the trail for further investigations on pregnancy-related conditions. As a novel biomarker in pregnancy-related diseases, PSP may provide new approaches for the management and discrimination of the severity of these conditions.

Hence, the aim of our study was to evaluate the levels of PSP in women with pathological pregnancies, such as PE, HELLP syndrome, PPROM, intraamniotic infection, or COVID-19 infection. The primary endpoint was the determination of serum PSP values in pregnant women with PE, HELLP syndrome, PPROM, intraamniotic infection, or COVID-19 infection, as well as its comparison to its norm values in pregnancy [13].

## 2. Material and Methods

*Study design and insight*

This prospective, single-centred cohort study evaluated the role of PSP as novel biomarker in women with pathological pregnancies. Pregnant women were recruited from 2013–2021.

*Study objectives*

Primary endpoint was the determination of serum PSP values in pregnant women with PE, HELLP syndrome, PPROM, intraamniotic infection, or COVID-19 infection, as well as its comparison to established norm values in pregnancy [13].

*Patients*

Inclusion criteria were age >18 years, women with the diagnosis of PE, HELLP syndrome, PPROM with or without intraamniotic infection (AIS), or COVID-19 infection, as well as written informed consent.

Exclusion criteria were pre-existing viral infections (Hepatitis B virus, Hepatitis C virus, human immunodeficiency virus).

PE was diagnosed in case of new onset of hypertension and proteinuria or new onset of hypertension and significant end-organ dysfunction (with or without proteinuria). Severe PE was defined as severe hypertension (systolic blood pressure ≥ 160 mmHg and/or diastolic blood pressure ≥ 110mmHg) and severe end-organ dysfunction (pulmonary edema, renal insufficiency, thrombocytopenia, impaired liver function, symptoms of central nervous system). HELLP syndrome was diagnosed if following criteria were fulfilled: hemolysis, elevated liver enzymes, and low platelets.

The diagnosis of a PPROM was made by maternal history, followed by a sterile speculum examination [14]. If no amniotic fluid was visualized leaking through the cervix, or if a vaginal pooling of amniotic fluid with a pH of 7 was observed, an AmniSure^®^ test was performed. This test detects the placental alpha microglobulin-1 protein (PAMG-1) with a PPROM sensitivity of 97.3% and specificity of 98.7% [15].

The diagnosis of intraamniotic infection was clinically suspected if pregnant woman was diagnosed with fever (≥ 39 °C on any one occasio, or temperature between 38–38.9 °C on repeated measurements), plus one or more of the following parameters: fetal tachycardia, elevated serum infection parameters (CRP or leucocytes), or purulent fluid from the cervical os.

The diagnosis of COVID-19 was confirmed by a professional nasopharyngeal PCR sampling.

Socioeconomic status was defined according to the parental education:

High—Higher education (university or comparable degree of both parents, or one parent with university degree and another with an apprenticeship degree).

Middle—Both parents with apprenticeship degree, or one parent with university degree and one who did not graduate.

Low—Both parents with no graduation, or one parent with apprenticeship and one who did not graduate.

*Materials/Sample collection and processing*

First, 5–10 mL of peripheral blood was drawn from each participant. Following arrival of the blood sample in the laboratory, serum PSP levels were measured by enzyme-linked immunosorbent assay (ELISA), as previously described [1,16].

Pregnant women were recruited at time of admission. They were assessed for eligibility to participate in the PSP clinical trial. Once the written informed consent form was understood and signed, blood samples were obtained and submitted for routine, as well as separate, analysis of PSP levels (one tube of 5–10 mL for serum determination in the laboratory). Blood samples were marked with patient’s name, date of birth, and date of sample extraction for identification during transportation to the laboratory. Following arrival of the blood sample in the laboratory, all patients’ information were pseudonymized. The clinical course was documented in the patient’s chart and added to the patient specific Case Report Form (CRF).

PSP analysis was performed using a validated Enzyme-Linked Immunosorbent Assay (ELISA) method described above [17]. All laboratory measurements were performed in triplicates. Excessive material was stored and catalogued in a central, locked biobank (at −80 °C temperature).

*Statistical methods*

Statistical significance was defined as *p* < 0.05. Results are expressed as mean ± standard deviation (SD), or median ± interquartile range (IQR) as appropriate, and were compared by Students *t*-test, ANOVA, or Wilcoxon’s rank sum test, as appropriate. Repetitive PSP measurements among groups were assessed by two-way ANOVA. Normal distribution of data was assessed with Shapiro–Wilk test. Categorical variables are presented as number (n) and percentage (%) and were compared with Fisher’s exact test. R V4.0.2 and R-Studio V1.3.1093 (R Foundation for Statistical Computing, Vienna, Austria) were used for statistical analyses, calculations, and graphical representations.

*Ethical approval*

This study was conducted with the principles of the Declaration of Helsinki [18] and International Conference on Harmonisation E6 (Good Clinical Practice) guidelines. The independent medical ethics committee of canton Zurich (Kantonale Ethikkommission, Zürich, Switzerland) has approved the study protocol (ID: KEK-ZH-Nr. 2014-0046). Swiss National Clinical Trials Portal SNCTP000000290; Institution Ethical Board Approval ID: KEK-ZH-Nr. 2014-0046. Clinicaltrials.gov ID: NCT02247297.

## 3. Results

In total, we included 152 pregnant women in the study. Eighty-four patients were diagnosed with PPROM, 53 with PE or HELLP syndrome, and 15 with COVID-19 infection. Intraamniotic infection was suspected in 31 (36.9%) women out of 84 with PPROM. Baseline characteristics of the different subgroups are shown in Table 1.

**Figure 1:**
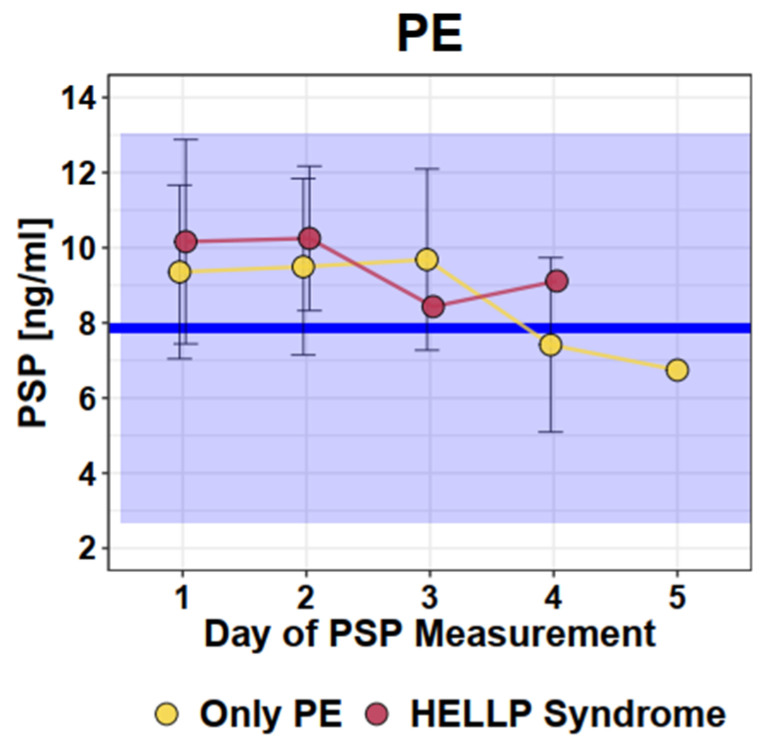
Longitudinal PSP values in women with pre-eclampsia/HELLP syndrome. Dots and error bars: Mean +/− SD; Blue line: Mean of healthy singleton pregnancy values; Blue square: 95% CI of all healthy singleton pregnancy values (Mean +/− 2 × SD).

Women who suffered from PE or HELLP syndrome displayed significantly increased PSP values at initial presentation (mean 9.8 ng/mL, SD 2.6) compared to normal singleton pregnant women (mean 7.9 ng/mL, SD 2.6, *p* ≤ 0.001). However, most measured values (90.6%) in women with PE/HELLP were within the normal range of PSP values of healthy pregnancies. Comparing patients with PE (mean 9.6 ng/mL, SD 2.5) and HELLP syndrome (mean 10.2 ng/mL, SD 2.7), no difference in repetitive PSP measurements up to Day 5 was observed: (*p* = 0.49). Graphical representation of the longitudinal PSP values in women with PE or HELLP syndrome is presented in Figure 1.

Concerning the severity of PE/HELLP, there were no cases of eclampsia in our study group. In our cohort, there were 18 women with HELLP syndrome and 15 women with severe PE. Twenty-nine of 53 (54.7%) women were admitted to an intermediate or intensive care unit on average for 2.4 days. In the HELLP syndrome group, there was one case with pulmonary oedema. Otherwise, there were no cases with liver hematoma, nor cases with renal impairment nor a need for dialysis in this cohort (highest Creatinine level in one patient was 103 µmol/L).

**Figure 2:**
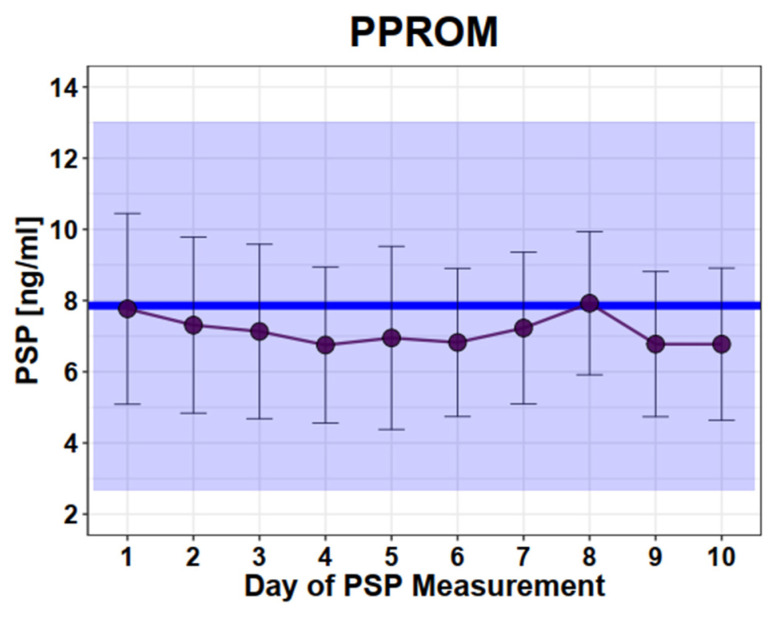
Longitudinal PSP values in women with PPROM. Legend: Dots and error bars: mean +/− SD; Blue line: mean of healthy singleton pregnancy values; Blue square: 95% CI of all healthy singleton pregnancy values (mean +/− 2 × SD). *y* axis: PSP values in ng/mL, *x* axis: days.

In pregnant women diagnosed with PPROM, we measured PSP values at different time points during the course of hospitalization. No difference of initial measured PSP values between women with PPROM and those with normal single pregnancies was observed (mean in PPROM: 7.9ng/mL versus mean in healthy 7.9 ng/mL, SD 2.6; SD 2.9, *p* = 0.98). Moreover, no significant change of the repetitive PSP measurements during hospitalization could be detected (Figure 2).

**Figure 3:**
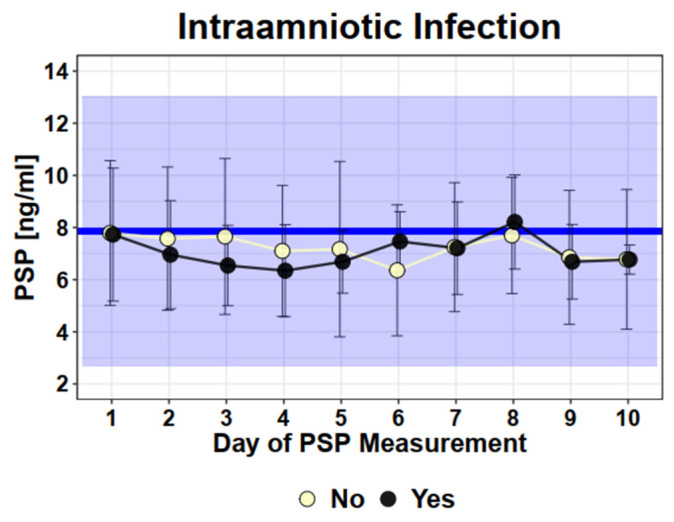
Longitudinal PSP values in PPROM patients with intraamniotic infection (yes) or without (no). Legend—Dots and error bars: Mean +/− SD; Blue line: Mean of healthy singleton pregnancy values; Blue Square: 95% CI of healthy singleton pregnancy values (Mean +/− 2 × SD).

To evaluate if PSP can discriminate women developing intraamniotic infection compared to those without intraamniotic infection after PPROM, we compared longitudinal measurements between these two groups (Figure 3). There was no difference in the PSP values in women with intra-amniotic infection versus women without intra-amniotic infection (infection: mean 7.9 ng/mL versus no infection: mean 7.8 ng/mL, SD 3.0), (SD2.8, *p* = 0.85).

**Figure 4:**
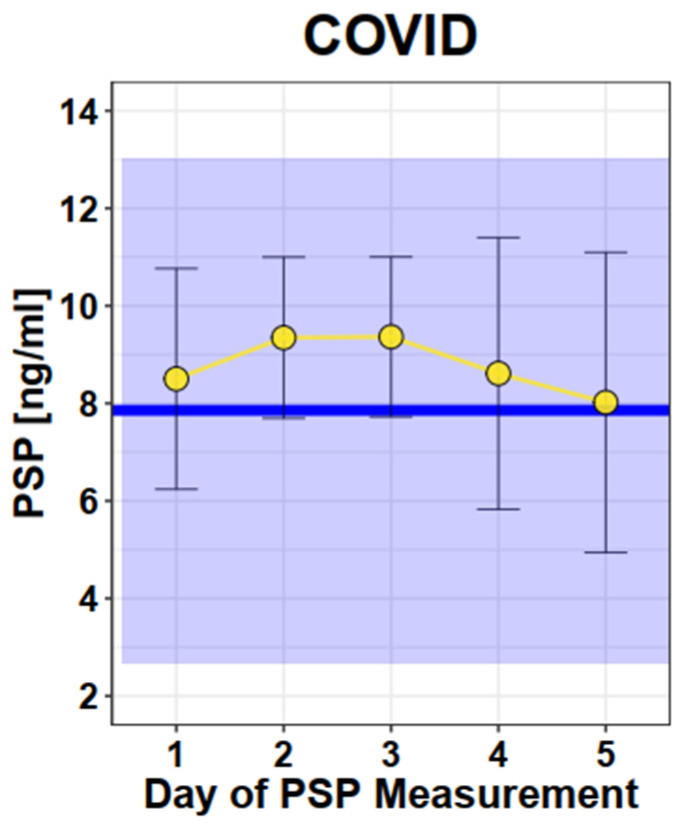
Longitudinal PSP values in COVID. Legend—Dots and Error bars: Mean +/− SD; Blue line: Mean of healthy singleton pregnancy values; Blue square: 95% CI of healthy singleton pregnancy values (Mean +/− 2 × SD).

The mean value of PSP in COVID-19-affected pregnant women was 8.5 ng/mL (SD 2.3). There was no significant change in the PSP value over the time of hospitalization (graphically shown in Figure 4). The PSP values of these women were not increased compared to normal healthy single pregnancies (*p* = 0.24).

Five COVID-19-positive pregnant women were hospitalized at an intensive care unit (*n* = 3) or intermediate care unit (*n* = 2). One patient was observed in an outpatient setting. Five patients suffered from COVID-19-associated pneumonia (mean value of their highest PSP value of the hospitalization: 10.1 ng/mL (SD 1.5)). Three of them were treated with antibiotics. Their PSP values were not increased compared to those without antibiotic treatment (mean: 9.0 ng/mL (SD 0.7). Seven needed oxygen therapy.

## 4. Discussion

### 4.1. Main Findings

This study evaluated the novel serum biomarker PSP in pregnancy-related diseases. We assessed PSP as a predictor of the severity of inflammatory and infectious complications in pregnant women. Significantly higher PSP values were observed in women diagnosed with PE or HELLP compared to the reference values in healthy women [13]. However, most measured values (90.6%) in women with PE/HELLP were within the normal range of PSP values of healthy pregnancies. However, for intra-amniotic infections after PPROM or COVID-19 infections, no significant changes in PSP levels were observed.

Reflecting the pathophysiology of the pregnancy-related medical conditions, which demonstrated evaluated PSP values in our study, PSP seems to be a promising biomarker for discriminating severity of PE or HELLP. Thus, several aspects deserve further consideration.

### 4.2. Interpretation

*PE/HELLP*

The pathophysiological pathways of PE or HELLP as systemic diseases with variable severities have not yet been entirely understood. However, increasing evidence suggests that a PE can lead to a widespread inflammation by an imbalance of angiogenic and antiangiogenic proteins [19]. As PSP was increased in women with PE, our results support the finding of a previous study that PSP might be a novel biomarker for the assessment of renal function in pregnant women [9]. In the study of Zhu et al., increased PSP/REG Iα concentrations were closely associated with renal dysfunction in pregnant women [9].

As we had no pregnant women with PE or HELLP with severe renal impairment, it might be possible that in very severe courses of these diseases, we might detect even much higher PSP values by activating a systemic inflammatory response of the pancreas and gut [1,3].

*PPROM/Intraamniotic infection*

PPROM is defined as a rupture of the membranes before the onset of uterine contractions and before 37 weeks of gestation, leading most often to preterm delivery, which may have adverse consequences for the physical and mental health of the child. Several risk factors seem to promote the occurrence of PPROM, such as ascending infections, cigarette smoking, and multiple pregnancy [20]. The management of women with PPROM differs depending on the gestational age and clinical situation (expectant versus delivery).

Acute chorioamnionitis, the histopathological counterpart of the clinical diagnosis of intraamniotic infection, is the most common pathological placental disorder and occurs in 50–60% of women with PPROM [21,22]. As chorioamnionitis poses a danger to the fetus and can promote the development of early onset sepsis and other neonatal morbidities, such as cystic periventricular leukomalacia and cerebral palsy, an early and accurate diagnosis of intra-amniotic infection is indispensable [23]. Moreover, acute chorioamnionitis predisposes the mother to develop sepsis, which might lead to organ failure and necessitate intensive care surveillance [24]. Serial measurements of CRP and leucocytes are often performed to discriminate the course of infection in women with PPROM. As PSP acts as an acute phase protein [2], we hypothesized it to be an additional marker for the discrimination of presence or absence of intra-amniotic infection in women with PPROM. Women with PPROM demonstrated no increase in PSP levels in comparison to the reference values of the healthy pregnant population. Further, PSP values did not allow for distinguishing women who developed intra-amniotic infection or not. A possible explanation of this result could be that at the beginning of the intra-amniotic infection, the source of infection is limited to the “semi-closed” system in the amniotic liquid, leading to uterus contractions (as a reaction of the body to fight against the infection focus). However, in some cases, the infection leads to a systemic pathophysiologic response, leading to sepsis and a life-threatening condition for the mother. In our cohort, no maternal transmission to an intermediate care or intensive care unit was necessary to demonstrate there were no severe cases of sepsis in this group. In other words, the delivery was performed before a severe maternal infection or before infection might have led to increased PSP values.

*COVID-19*

During the current COVID-19 pandemic, PSP has proven to be an excellent parameter, in combination with the respiratory sequential organ failure assessment score (cSOFA), to assess the severity of the clinical course of patients suffering from infectious diseases, as well as to facilitate the further triage for therapeutic strategy of these patients [25].

In COVID-19 patients, PSP seems to be a potential biomarker of bacterial infections [26]. However, PSP values in pregnant women diagnosed with COVID-19 have not been reported so far. In a proof-of-concept approach, we included 15 pregnant women with COVID-19.

Our PSP values in COVID-19-affected pregnancies were in line with the normal pregnant (LIT) and non-pregnant cohort [13].

They also correspond with the recently published data of Van Singer et al. [27], evaluating the role of PSP in non-pregnant COVID-19 patients for early mortality. PSP appeared to be a good biomarker to exclude short-term risk of death, but not to exclude ICU admission in the context of COVID-19. They conclude that there might be another pathophysiological pathway at the bottom of the infection in COVID-19 (e.g., compared to sepsis of gastrointestinal origin).

### 4.3. Strengths and Limitations

An advantage of our study is that the biomarker PSP was evaluated not only in one, but in several pregnancy-related pathologies, such as PE, HELLP syndrome, PPROM, and COVID-19 for the first time. Data were prospectively collected and standardized, and reproducible methods were used.

In this proof-of-concept study, we demonstrated that PSP is a potential biomarker in PE or HELLP syndrome. As only mild-to-moderate cases with renal insufficieny or liver hematoma were included in our study, we might detect even higher PSP values in more severe cases, which might then, in time, be used for an accurate treatment. Therefore, these first results allow for the further randomization of controlled studies to confirm our findings.

## 5. Conclusions

PSP might be a promising marker in PE and HELLP syndrome. These observations remain to be confirmed in further prospective randomized trials.

## Figures and Tables

**Table 1:** Baseline characteristics of women with PE/HELLP, PPROM, and COVID-19 infection.

	PE/HELLP (N = 53)	PPROM (N = 84)	COVID-19 (N = 15)	Total (N = 152)
**Maternal Age (years)**				
Median [Q1, Q3]	34.0 [31.0, 37.0]	33.0 [28.0, 37.0]	34.0 [26.0, 36.0]	33.0 [29.0, 37.0]
**Gestational age (weeks)**				
Median [Q1, Q3]	33.8 [30.0, 37.0]	31.7 [26.2, 34.4]	30.0 [27.5, 33.5]	32.1 [27.9, 35.7]
Missing	1.00 (1.9%)	1.00 (1.2%)	0 (0%)	2.00 (1.3%)
**Body mass index (BMI) (kg/m^2^)**				
Median [Q1, Q3]	23.0 [21.0, 26.3]	22.9 [21.3, 26.5]	26.6 [22.1, 31.4]	23.0 [21.1, 27.2]
**Ethnicity**				
Afro Caribbean	1 (1.9%)	2 (2.4%)	5 (33.3%)	8 (5.3%)
Asian	2 (3.8%)	3 (3.6%)	0 (0.0%)	5 (3.3%)
Caucasian	44 (83.0%)	59 (70.2%)	9 (60.0%)	112 (73.7%)
Mediterranean	2 (3.8%)	6 (7.1%)	1 (6.7%)	9 (5.9%)
Mixed	0 (0.0%)	1 (1.2%)	0 (0.0%)	1 (0.7%)
Oriental	0 (0.0%)	5 (6.0%)	0 (0.0%)	5 (3.3%)
Missing	4.00 (7.5%)	8.00 (9.5%)	0 (0%)	12.0 (7.9%)
**Socioeconomic status**				
High	8 (15.1%)	17 (20.2%)	2 (13.3%)	27 (17.8%)
Low	20 (37.7%)	25 (29.8%)	2 (13.3%)	47 (30.9%)
Middle	22 (41.5%)	39 (46.4%)	10 (66.7%)	71 (46.7%)
Missing	3.00 (5.7%)	3.00 (3.6%)	1.00 (6.7%)	7.00 (4.6%)
** Initial first measured PSP value (ng/mL)**				
Mean (SD)	9.82 (2.58)	7.86 (2.89)	8.50 (2.26)	8.60 (2.86)

## Data Availability

Data are available in the main text.

## Abbreviations

AIRS	Amniotic inflammatory response syndrome
COVID-19	Coronavirus disease 2019
CRF	Case report form
CRP	C-reactive protein
ELISA	Enzyme Linked Immunosorbent Assay
HELLP	Hemolysis, elevated liver enzymes, and low platelets
IL-6	Interleukin 6
ICU	Intensive care unit
PCT	Procalcitonin
PPROM	Preterm premature rupture of membranes
PSP	Pancreatic stone protein
SNCTP	Swiss National Clinical Trials Portal
SOFA	Sequential organ failure assessment
